# A case report and literature review: *Mycobacterium leprae* infection diagnosed by metagenomic next-generation sequencing of cerebrospinal fluid

**DOI:** 10.1186/s12879-024-09473-z

**Published:** 2024-07-03

**Authors:** Conglin Zhao, Zhenzhen Liu

**Affiliations:** Center of Infectious Diseases, West China Hospital, Sichuan University, 37 Guoxue Lane, Chengdu, 610041 China

**Keywords:** *Mycobacterium leprae*, Leprosy, *Listeria monocytogenes*, Metagenomic next-generation sequencing, Systemic lupus erythematosus, Case report

## Abstract

**Background:**

Leprosy is a chronic infectious disease caused by *Mycobacterium leprae* (*M. leprae*) that is responsible for deformities and irreversible peripheral nerve damage and has a broad spectrum of clinical and serological manifestations. Leprosy primarily affects the peripheral nerves and rarely presents with central nervous system involvement. Diagnosing leprosy can still be difficult in some cases, especially when the infection involves uncommon clinical manifestations and extracutaneous sites. Delayed diagnosis and treatment of leprosy may lead to irreversible damage and death.

**Case Presentation:**

We report a case of a 30-year-old female presenting with “repeated high fever with symptoms of headache for 14 days”. On the day of admission, physical signs of lost eyebrows and scattered red induration patches all over her body were observed. The patient’s diagnosis was based on the clinical characteristics using a combination of metagenomic next-generation sequencing (mNGS) of cerebrospinal fluid (CSF) and slit-skin smear. After confirming Listeria meningitis and multibacillary leprosy with erythema nodosum leprosum (ENL), a type 2 reaction, she was treated with ampicillin sodium, dapsone, rifampicin, clofazimine, methylprednisolone, and thalidomide. At the 1-year follow-up, the frequency and severity of headaches have significantly decreased and a good clinical response with improved skin lesions was found.

**Conclusion:**

This case highlights the importance of considering leprosy, which is a rare and underrecognized disease, in the differential diagnosis of skin rashes with rheumatic manifestations, even in areas where the disease is not endemic, and physicians should be alerted about the possibility of central nervous system infections. In addition, mNGS can be used as a complementary diagnostic tool to traditional diagnostic methods to enhance the diagnostic accuracy of leprosy.

## Background

Leprosy, also known as Hansen’s disease, is a chronic bacterial infection caused by *Mycobacterium leprae* (*M. leprae*) that primarily affects the skin and peripheral nerves and is mainly transmitted by respiratory droplets [42]. Although the incidence rate of leprosy presents a declining trend each year, it remains a significant public health problem [12]. It has low virulence and an average incubation period of 4–5 years with a broad spectrum of clinical and serological manifestations [37]. According to the World Health Organization (WHO) diagnostic criteria, if a patient has one of the two cardinal signs present, (1) a positive skin smear or (2) characteristic cutaneous leprosy skin lesions with or without thickening/enlargement of the nerve associated with loss of sensation or motor function, they are considered to have leprosy [7].

According to the the number of skin lesions, the patients of leprosy can be classified as paucibacillary (PB) and multibacillary (MB) leprosy cases [33]. The recommended treatment for leprosy includes rifampicin, dapsone, clofazimine, ofloxacin, minocycline, and vaccines. In addition, leprosy is associated with autoantibodies, and its clinical features are similar to connective tissue diseases [5, 21, 9]. Due to the complex and nonspecific clinical manifestations of leprosy, it can be easily overlooked or misdiagnosed, resulting in delayed treatment and more severe complications. Therefore, early diagnosis and prompt initiation of treatment are crucial to prevent the development of irreversible damage and disability [41].

Leprosy reactions are severe and potentially life-threatening conditions that can occur in leprosy patients during or after treatment. These reactions typically result from a sudden imbalance in the immune system that has developed during the course of the disease, in response to Mycobacterium leprae [19]. They can be divided into type 1, type 2 (Erythema Nodosum Leprosum, ENL), and type 3 (Lucio phenomenon) reactions [1]. ENL is considered an immune complex (IC)-mediated reaction that can occur before, during, or after the course of multidrug therapy (MDT), which might be triggered by parturition, pregnancy, and pyrogenic infection [23, 40]. Clinical manifestations may include fever, cutaneous and subcutaneous erythematous nodules, arthritis, lymphadenopathy, neuritis, iridocyclitis, glomerulonephritis, and hepatitis [23, 16]. Thalidomide and corticosteroids are the primary treatment options for ENL [13].

Metagenomic next-generation sequencing (mNGS) is a new high-throughput detection method based on the next-generation sequencing (NGS) platform [47]. It demonstrates a relatively unbiased and compassionate approach to simultaneously detecting hundreds of pathogens, including emerging pathogens. This presents great potential for analyzing pathogens in clinical specimens, aiding in diagnosing and treating various infectious diseases [43]. At the same time, improving mNGS technology has shown broad application prospects in diagnosis of infectious diseases [11]. Many studies have proven the great potential of mNGS in infectious disease diagnostics, especially for new, rare, and mixed infections [27, 45, 35].

In this study, we highlighted the diagnosis and treatment process of a case of leprosy, providing valuable insights into this often overlooked disease. The information presented may encourage physicians to consider leprosy more frequently as a potential differential diagnosis, ultimately leading to more accurate diagnoses and improved treatment outcomes.

## Case Presentation

The patient was a 30-year-old female admitted to the hospital with repeated high fever with symptoms of headache for 14 days. She had no apparent cause of fever or headache for 14 days before admission, with a maximum temperature of 39.6 °C, no joint aches and photosensitivity, no hypoesthesia of the hands or feet, and no chest tightness, shortness of breath, abdominal distension, or pain. Contrast-enhanced brain magnetic resonance imaging (MRI) showed dural thickening and diffuse leptomeningeal enhancement in the cerebellar vermis, and this lesion was thought to be meningitis. Cerebrospinal fluid and blood cultures showed *Listeria monocytogenes*, so Listeria meningitis and bacteremia were diagnosed. She received penicillin therapy at the First People’s Hospital of Yunnan Province. Although her body temperature had returned to normal, the patient’s headaches did not subside significantly. As a result, she was transferred to our hospital for further treatment.

The patient had been diagnosed with chronic hepatitis B over five months ago and had been consistently taking oral antiviral therapy with entecavir. She had been diagnosed with systemic lupus erythematosus (SLE) 5 months ago when she presented with skin rashes and diffuse erythema nodosum lesions on the trunk. The patient was treated with prednisone and hydroxychloroquine (details unavailable). Furthermore, she reported no recent travel history, surgery, or trauma and no notable medical or family history.

**Fig. 1:**
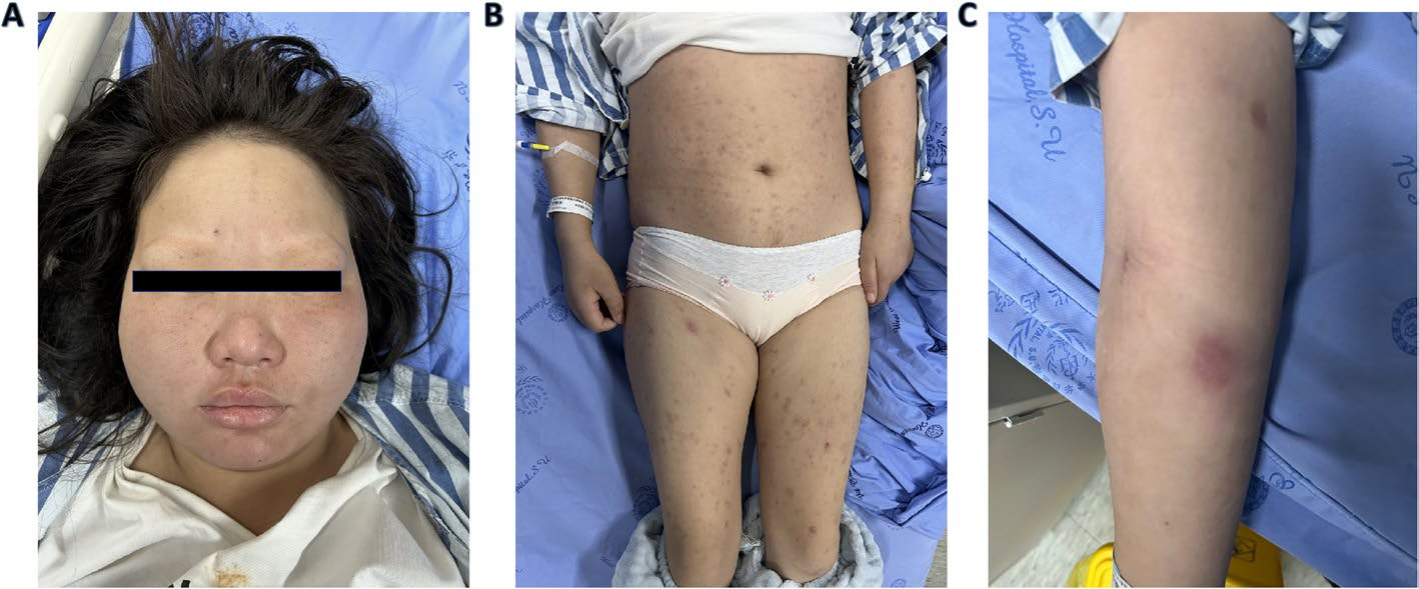
Photographs of the patient. **A** The eyebrows of the patient were lost. **B** Erythematous lesions on the trunk and extremities. **C** Erythematous nodules on the right arm

The patient was referred to the Rheumatology Department. She had no fever (36.3 °C) at admission, and routine physical examinations were conducted, which included measuring the patient’s pulse rate (80/min), respiratory rate (18/min), blood pressure (108/65 mmHg), and oxygen saturation (99%). On the day of admission, the loss of eyebrows (Fig. 1a) and scattered patches of red induration all over her body were observed (Fig. 1b). Scattered firm red skin nodules emerged in the right upper limb and lower extremity (Fig. 1c). The rest of the patient’s physical examination revealed no notable findings.

**Fig. 2:**
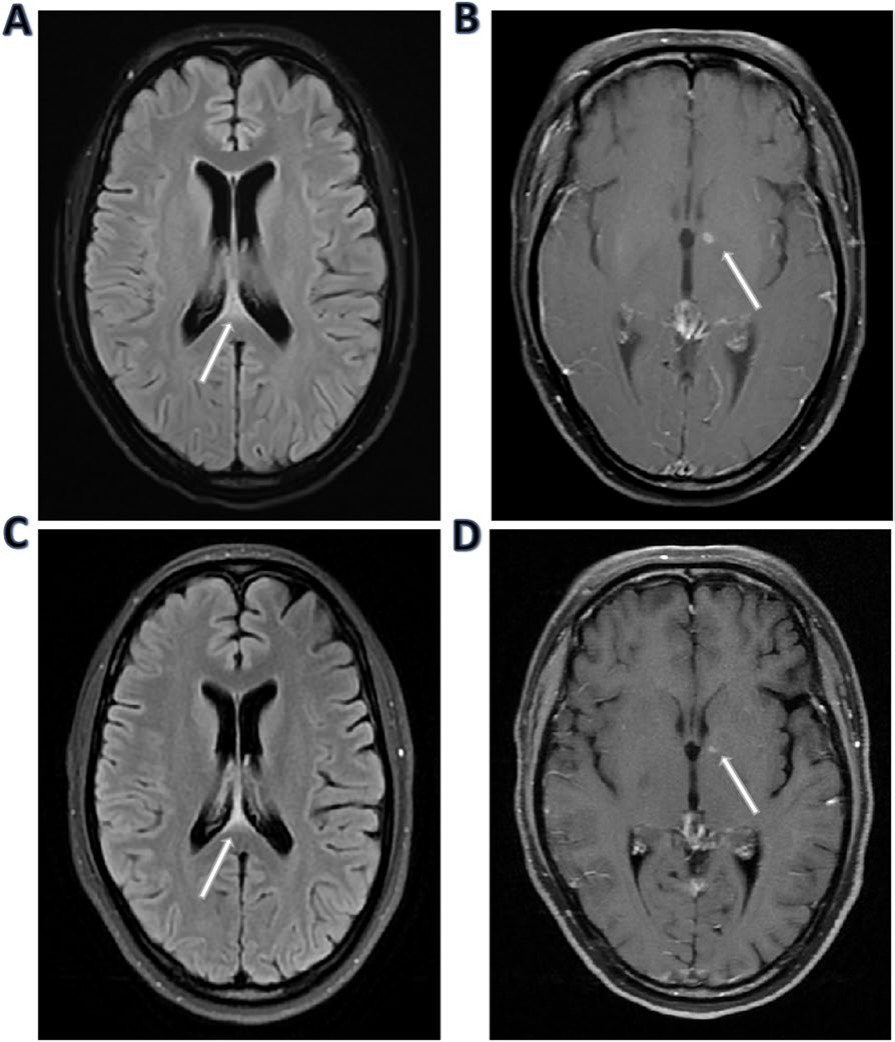
Brain MRI results. **A** A few enhancement lesions on the upper edge of the cerebellar tonsil. **B** The dominant enhancement in the left basal ganglia is probably infection foci. **C, D** A decrease in the upper edge of the cerebellar tonsil and left basal ganglia lesion size

Laboratory investigations (Table 1) showed decreased hemoglobin (108 g/L, normal range, 115–150 g/L), albumin (33.3 g/L; normal range, 40.0–55.0 g/L), CD4 cell count (285 cell/µL; normal range, 471–1220 cell/µL), B-cell count (117 cell/µL; normal range, 175–332 cell/µL), natural killer (NK) cell count (12 cell/µL; normal range, 154–768 cell/µL), complement C3 (0.7050 g/L; normal range, 0.785–1.520 g/L), and C4 (0.1240 g/L; normal range, 0.145–0.360 g/L). The patient’s white blood cells [11.43 × 109/L; normal range, (3.5–9.5)×10^9^/L], procalcitonin level (0.26 g/L; normal range, < 0.046 ng/L), C-reactive protein (7.85 ng/L; normal range, < 5.00 ng/L), interleukin-6 (9.95 pg/ml; normal range, 0.00–7.00 pg/ml), erythrocyte sedimentation rate (ESR) (59.0 mm/h; normal range, < 26 mm/h), immunoglobulin IgM (2300.00 mg/L; normal range, 700–2200 mg/L), rheumatoid factor (32.10 IU/mL; normal range, < 20.00 IU/mL), anticardiolipin IgA antibody (> 120.00 APLU/mL; normal range, < 10 APLU/mL), anticardiolipin antibody IgM (> 120.00 MPLU/mL; normal range, < 10 APLU/mL), anti-β2-glycoprotein antibodies IgA (384.00 AU/mL; normal range, < 20 AU/mL), anti-β2-glycoprotein antibodies IgM (> 841.00 AU/mL; normal range, < 20 AU/mL) and HBV-DNA (1.45E + 4) levels were elevated. The patient’s platelet [252 × 10^9^/L; normal range, (100–300)×10^9^/L], globulin (28.8 g/L; normal 20–35 g/L), triacylglycerol (2.94 mmol/L; normal range, 0.29–1.83 mmol/L), fibrinogen (3.72 g/L; normal 2.0–4.0 g/L) and serum galactomannan levels (0.06; normal range, < 0.5) were in the normal ranges. Tests for HCV-RNA, CMV-DNA, EB-DNA, HIV-1 antibody, anti-tuberculosis antibody, TB-IGRA, and 1,3-β-D-glucan peripheral blood cultures drawn were negative. Antinuclear antibody (Ana) was positive. Immunoglobulin IgG and IgA, antineutrophil cytoplasmic antibody ANCA, anti-double-stranded DNA antibody, anti-SM antibody, anti-SSA, SSB antibody, anti-Ro52 antibody, anti-ScL-70 antibody, anti-Jo-1 antibody, anticardiolipin antibody IgG and anti-β2-glycoprotein antibodies IgG were also normal, and there was no abnormality in urination and stool tests. Brain MRI shows a few enhancement lesions on the upper edge of the cerebellar tonsil and dominant enhancement in the left basal ganglia, probably infection foci (Fig. 2a, b).

**Table 1:** Blood Test Results

Inspection Items	Day1	Day3	Day7	Day14	Day 18	Reference Values
Hemoglobin (Hb) (g/L)	108	95	99	84	83	115–150
Red blood cell (RBC) count (×10^12^/L)	4.20	3.60	3.70	3.15	3.12	3.8–5.1
White blood cell count (×10^9^/L)	11.43	9.67	7.79	4.79	3.40	3.5–9.5
Platelets (×10^9^/L)	252	153	121	96	161	100–300
C-reactive protein (mg/L)	7.85	14.00	28.10	132	47.40	< 5.00
IL-6 (pg/mL)	9.95	9.42	12.20	33.90	16.80	0.00–7.00
Procalcitonin (ng/mL)	0.26	0.30	0.26	0.55	0.35	< 0.046
Glucose levels(mmol/L)	5.95	5.33	4.87	5.40	4.92	3.90–5.90

*Abbreviation*: *IL-6*, interleukin 6

After admission, we administered ampicillin sodium (2 g every four hours), methylprednisolone (40 mg once a day), and antiviral therapy with entecavir (0.5 mg once a day). For further evaluation, a lumbar puncture was performed. The cerebrospinal fluid (CSF) was clear, with elevated trace protein levels (1.92 g/L) and IgG synthesis rates (56.319 mg/day). There were no abnormalities in nucleated cell count (6 × 10^6^/L), glucose level (4.36 mmol/L), smear, or culture of CSF (Table 2). At the same time, mNGS was performed. mNGS was performed according to the standard protocol of Illumina sequencing on the NextSeq550 platform. A total of 667 sequence reads of *Listeria monocytogenes* and 272 sequence reads of *Mycobacterium leprae* were detected in CSF, accounting for 0.25% and 0.08% of the genome coverage, respectively.

**Table 2:** Cerebrospinal fluid analysis

CSF Results	Day 1	Day 7	Day 18	Reference Values
CSF pressure (mmH_2_O)	90	108	130	80–180
Nucleated cell count (×10^6^/L)	6	15	12	0–10
Trace protein (g/L)	1.92	1.03	0.57	0.15–0.45
Glucose levels (mmol/L)	4.36	2.60	2.78	2.5–4.4
Chloride levels (mmol/L)	114	118	123	120–130
Ink-stained	negative	negative	negative	negative
Cryptococcal antigen titer	negative	negative	negative	negative
Gram staining	negative	negative	negative	negative
Acid-fast staining	negative	negative	negative	negative
CSF culture	negative	negative	negative	negative
Real-time fluorescence detection of Mycobacterium tuberculosis DNA			negative	negative
IgG synthesis rates (mg/day)	56.319	32.652	14.283	0.0-5.81
Metagenomic Next-generation Sequencing	*Listeria monocytogenes* (667 sequence reads); *Mycobacterium leprae* (272 sequence reads)	*Listeria monocytogenes* (163 sequence reads); *Mycobacterium leprae* (39 sequence reads)	*Mycobacterium leprae* (322 sequence reads)	
Pathological results			nonmalignant cells	

1 mmH_2_O = 0.0098 kPa

Based on these findings, she was transferred to our center because *Mycobacterium leprae* infection was highly suspected. On further clinical interrogation of the patient, she had suffered from scattered skin rashes without pain or itch and loss of eyebrows for three years. Unfortunately, she gave little importance to these symptoms. We suspect she has leprosy based on erythematous lesions, loss of eyebrows, and the result of *M. leprae* in CSF. To confirm this hypothesis, we further performed a slit-skin smear. From the right eyebrow orbit, the right earlobe, jaw, right elbow, left elbow, and right knee showed *leprae* positivity, with an average bacterial index (BI) of 4.83, representing a high number of bacilli.

The clinical diagnosis was listeria meningitis and multibacillary leprosy (MB) with erythema nodosum leprosum (ENL), a type 2 reaction [13, 2, 44]. Due to a type 2 reaction state, presenting with erythematous nodules, multidrug therapy (MDT) with rifampicin (600 mg once a day), dapsone (100 mg once a day), clofazimine (100 mg once a day), thalidomide (50 mg 3 times a day) and a tapered off of the corticosteroids was initiated. Repeat lumbar punctures were performed on day seven postadmission. A total of 163 sequence reads of *Listeria monocytogenes* and 39 sequence reads of *Mycobacterium leprae* were again detected in CSF.

After treatment, her headache symptoms improved. On the 18th day after admission, mNGS of CSF did not detect *Listeria monocytogenes*, while *Mycobacterium leprosy* was still detected (322 sequence reads). On the 26th day after admission, MRI contrast-enhanced scanning showed a decrease in the upper edge of the cerebellar tonsil and left basal ganglia lesion size (Fig. 2c, d). She was discharged on day 29 of admission. Our patient developed neuropathic pain in both lower extremities, and her skin nodules and headache improved during the 1-year follow-up.

## Discussion and conclusions

This case introduces the diagnosis and treatment process of a case of leprosy misdiagnosed as SLE for a long time. Leprosy is a chronic infectious disease caused by *M. leprae* [34]. It remains a worldwide public health problem, despite significantly reducing its prevalence over time [36]. In recent years, the diagnosis methods of leprosy included a slit-skin smear test, pathological examination, and molecular approaches, such as PCR [3]. Due to its nonspecific and complex clinical manifestations, leprosy can be easily overlooked. A high degree of suspicion is the key to early diagnosis of leprosy [10]. Delayed diagnosis and treatment of leprosy may lead to irreversible damage and death.

Leprosy classically presents with skin and neurological manifestations [24]. The symptoms of leprosy are often nonspecific and vary because of bacterial load and different host immune responses [19]. There are similarities between clinical symptoms and serological tests in patients with leprosy and connective tissue disorders, which might delay early recognition and diagnostic confusion. In addition, the coexistence of leprosy with SLE has also been reported [21]. The most common rheumatologic symptoms include inflammatory polyarthritis, skin rash, ulcers, and gangrene [21, 24]. There are some serological similarities between autoimmune diseases and leprosy, including antinuclear antibodies (ANA), antinuclear cytoplasmic antibodies (ANCA), rheumatoid factor (RF), antineutrophil cytoplasmic antibody, anti-cyclic citrullinated peptide, and antiphospholipid antibodies [32, 46]. Among leprosy and chronic inflammatory diseases, the exact pathogenesis and mechanism of the generation of autoantibodies remain elusive. It is therefore crucial for clinicians to recognize the possible link between rheumatic symptoms and leprosy, as delayed diagnosis leading to delayed treatment initiation could result in worse outcomes.

However, little is understood about the involvement of the CNS in leprosy. Leprosy primarily affects the peripheral nerves, mainly the posterior tibial nerve and the ulnar, median, and lateral popliteal, and rarely presents with central nervous system (CNS) involvement [6, 39]. Peripheral nerves involvement in leprosy affects the sensory, motor, and autonomic function, progressively leading to impairments and disability [22].

First, the blood-brain barrier (BBB) plays a vital role in maintaining the normal physiological function of the CNS [20]. The BBB is compromised in many chronic infections [30]. In leprosy, inflammation is common during reactions, and millions of bacilli are present in the blood and tissues [17].There is a possibility that leprosy bacilli or their products can cross the host’s BBB and cause pathological nerve changes [22]. Some studies using sensitive immunoassays and immunoblotting techniques that rely on monoclonal antibodies have also detected *M. leprae* antigens in the CSF of leprosy patients [38]. Experimental studies have shown that *M. leprae* can cross the BBB in mice that have undergone thymectomy and radiation. It can infiltrate the brain of ordinary mice [38]. In both cases, bacilli were detected in brain tissue sections. Most of these studies found bacilli in the spinal cord, dorsal root, cranial nerve ganglia, and Purkinje cells of the cerebellum [28, 18].

Second, abnormalities in neuroimaging of the spinal cord, brain and brainstem have also been reported in some leprosy cases [4, 29, 25]. Generally, the facial and trigeminal nerves are leprosy’s most commonly involved cranial nerves [26]. A study reported 8 cases of leprosy involving the CNS [29]. Two patients were discovered to have brainstem lesions, with one displaying enhanced facial nuclei and nerves, while the other had an ambiguous lesion in the nucleus. Furthermore, severe cases have been found to exhibit abnormalities in brainstem auditory-evoked potentials, suggesting that the brainstem can also be impacted [8]. A prospective observational study screened 54 patients diagnosed with multibacillary leprosy through bacteriological confirmation [39]. Five patients (17.24%) had abnormal findings in the CNS and spinal root ganglion as determined by MRI. In one patient, T2/FLAIR hyperintensity was observed in the middle cerebellar peduncle. Except for one patient with hyperreflexia of the lower limbs, none with central nervous system involvement exhibited any clinical symptoms related to brain or spinal cord involvement.

There are three potential explanations for the MRI anomalies observed in leprosy: (1) retrograde dissemination of *M. leprae* via peripheral nerves, nerve roots, and into the spinal cord; (2) responsive alterations in the spinal cord induced by sectioning of axons from peripheral nerves or roots; and (3) an immunological reaction to the bacterial antigen as the most probable cause of CNS involvement, according to study [29].

In our case, we successfully detected multiple *M. leprae* from CSF by mNGS, while the acid-fast bacilli staining of the CSF was negative. Brain MRI shows a few enhancement lesions on the upper edge of the cerebellar tonsil and dominant enhancement in the left basal ganglia. It differs from the previously reported location of lesions involving the CNS of *M. leprae* in MRI. As the patient had concomitant Listeria meningitis, we could not observe the definite characteristics of intracranial *M. leprae* infection. Multidrug therapy (MDT) is currently recommended in leprosy patients [29]. However, reports of clofazimine have shown low CSF penetration [14, 15]. The treatment options for pathological mechanisms, clinical characteristics, and optimal intracranial *M. leprae* infection remain unclear. Therefore, physicians should be alerted about the possibility of CNS infections due to *M. leprae*. More investigations are needed to elucidate this rare condition.

mNGS is a hypothesis-free, method that has been developed and optimized for clinical detection of multiple pathogens, especially unculturable ones, and can be helpful in the differential diagnosis of low-incidence infectious diseases [31]. The advantages of mNGS were confirmed in our case. In our case, mNGS of CSF revealed M. leprae and Listeria monocytogenes infections. This result provided additional clues that *M. leprae* could induce skin rash in the patient. Then, we further performed slit-skin smears, confirming the leprosy diagnosis. This case illustrated that mNGS might offer valuable insights for clinicians who may encounter cases in nonendemic areas less frequently. Testing the CSF can provide important diagnostic information when assessing leprosy patients with neurologic complications.

Here, we report a rare case of M. leprae and Listeria monocytogenes DNA detection from CSF using mNGS which further guided us to perform a skin smear and enabled us arrive at a definite diagnosis of leprosy. Due to a lack of knowledge or low clinical suspicion of leprosy, delayed diagnosis and proper treatment in our patients may be common in nonendemic areas. There is a need to raise awareness among physicians about leprosy to initiate appropriate treatment more rapidly, and physicians should be alerted about the possibility of CNS infections due to *M. leprae*. In addition, understanding the neurologic complications of this infection is crucial for doctors to diagnose and treat the disease better. Using mNGS as a diagnostic tool can improve the accuracy of leprosy diagnosis.

## Data Availability

The clinical records of the patient are available from the corresponding author on reasonable request. The sequencing data have bee in the NCBl Sequence Read Archive (SRA) database under the accession code SRR28580487 [https://www.ncbi.nlm.nih.gov/sra/?term=SRR28580487] and SRR28592923 [https://www.ncbi.nlm.nih.gov/sra/?term=SRR28592923].

## Abbreviations

CSF: Cerebrospinal fluid
ENL: Erythema nodosum leprosum
MDT: Multidrug therapy
NGS: Next-generation sequencing
MRI: Magnetic resonance imaging
SLE: Systemic lupus erythematosus
CNS: Central nervous system
BBB: Blood-brain barrier
